# A small molecule inhibitor of atypical protein kinase C signaling inhibits pancreatic cancer cell transformed growth and invasion

**DOI:** 10.18632/oncotarget.3812

**Published:** 2015-04-14

**Authors:** Amanda M. Butler, Michele L. Scotti Buzhardt, Eda Erdogan, Shuhua Li, Kristin S. Inman, Alan P. Fields, Nicole R. Murray

**Affiliations:** ^1^ Department of Cancer Biology, Mayo Clinic, Jacksonville, FL, USA; ^2^ Genoptix/Novartis Molecular Diagnostics, Carlsbad, CA, USA; ^3^ Department of Biomedical Sciences and Pathobiology, Virginia Polytechnic Institute and State University, Blacksburg, VA, USA

**Keywords:** atypical PKCs, aurothiomalate, pancreatic cancer, transformed growth, invasion

## Abstract

Pancreatic cancer is highly resistant to current chemotherapies. Identification of the critical signaling pathways that mediate pancreatic cancer transformed growth is necessary for the development of more effective therapeutic treatments. Recently, we demonstrated that protein kinase C iota (PKCι) and zeta (PKCζ) promote pancreatic cancer transformed growth and invasion, by activating Rac1→ERK and STAT3 signaling pathways, respectively. However, a key question is whether PKCι and PKCζ play redundant (or non-redundant) roles in pancreatic cancer cell transformed growth. Here we describe the novel observations that 1) PKCι and PKCζ are non-redundant in the context of the transformed growth of pancreatic cancer cells; 2) a gold-containing small molecule known to disrupt the PKCι/Par6 interaction, aurothiomalate, also disrupts PKCζ/Par6 interaction; 3) aurothiomalate inhibits downstream signaling of both PKCι and PKCζ, and blocks transformed growth of pancreatic cancer cells *in vitro*; and 4) aurothiomalate inhibits pancreatic cancer tumor growth and metastasis *in vivo*. Taken together, these data provide convincing evidence that an inhibitor of atypical PKC signaling inhibits two key oncogenic signaling pathways, driven non-redundantly by PKCι and PKCζ, to significantly reduce tumor growth and metastasis. Our results demonstrate that inhibition of atypical PKC signaling is a promising therapeutic strategy to treat pancreatic cancer.

## INTRODUCTION

Pancreatic cancer remains the fourth leading cause of cancer deaths in the United States [1]. Pancreatic cancer has a high propensity for resistance to current chemotherapy treatments; thus molecular targeting of multiple pathways necessary for tumor cell growth and maintenance may be required for successful therapeutic intervention. Recently, we demonstrated a role for the atypical protein kinase C (aPKC) isozymes, PKCι and PKCζ, in the transformed growth and invasive phenotype of pancreatic cancer cells [2, 3]. Aurothiomalate (ATM) forms a thio-gold adduct with a cysteine residue within the Phox-Bem1 (PB1) domain of PKCι (Cys69), thereby blocking PB1 domain-mediated interactions with components of PKCι's pro-cancer signaling complex, and inhibiting PKCι oncogenic function [4]. Similar to genetic inhibition of PKCι, ATM inhibits soft agar colony formation of lung cancer cells *in vitro* and orthotopic lung tumor growth and proliferation *in vivo* [4-6]. Auranofin (ANF), a second small molecule gold-containing compound in the same chemical class as ATM, inhibits PKCι signaling in ovarian cancer, via a mechanism of action similar to that of ATM [7]. PKCζ is structurally similar to PKCι and also contains a cysteine residue in the protein-binding region of the PB1 domain (Cys68); therefore these gold-containing compounds are predicted to disrupt PKCζ-mediated downstream signaling.

In the present study, we show that PKCι and PKCζ play non-redundant, required roles in pancreatic cancer cell transformed growth. We demonstrate for the first time that ATM disrupts binding of PKCζ to partitioning defective 6 homolog (Par6), an aPKC signaling partner, and that inhibition of expression of Par6 significantly reduces transformed growth and invasion of pancreatic cancer cells, suggesting that aPKC/Par6 signaling is an important mediator of the transformed phenotype of pancreatic cancer cells. Furthermore, ATM inhibits the transformed growth of pancreatic cancer cell *in vitro* and tumor formation *in vivo*, demonstrating the therapeutic potential of disrupting aPKC signaling.

## RESULTS

### PKCι and PKCζ play non-redundant roles in the transformed phenotype of pancreatic cancer

**Figure 1:**
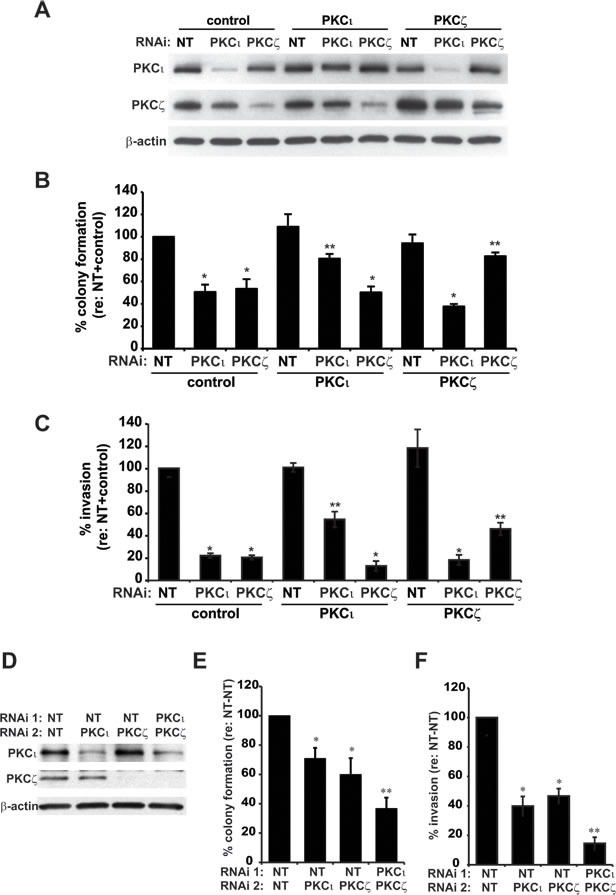
PKCι and PKCζ play non-redundant roles in pancreatic cancer cell transformed growth **A-C**) Panc-1 cells transfected with lentivirus expressing either control, non-targeting (NT) RNAi, PKCζ-targeting RNAi, or PKCι-targeting RNAi, and also carrying either control vector (pBabe), or vector expressing PKCι or PKCζ were assayed for A) expression of PKCι, PKCζ and actin by immunoblot analyses, **B**) anchorage-independent growth (colony formation in soft agar), and **C**) cellular invasion through Matrigel-coated transwell chambers. **B, C**) Bars = average of 3 or more replicates±SD and graph is representative of 2 or more independent experiments.*p < 0.05 vs NT-pBabe; **p < 0.05 vs corresponding RNAi + control vector. **D-F**) Panc-1 cells transfected with two doses of NT RNAi (control), NT and PKCι RNAi, NT and PKCζ RNAi, or PKCι and PKCζ RNAi were assessed for **D**) protein expression by immunoblot analysis, **E**) anchorage-independent growth (colony formation in soft agar), and **F**) cellular invasion. **E, F**) Data are expressed relative to NT+control±SD, n=3 or more, and graph is representative of 2 or more independent experiments. *p < 0.05 *vs* NT-NT; **p < 0.05 vs corresponding NT+ PKCι or NT+ PKCζ RNAi cell lines.

We have previously shown that inhibition of expression of either PKCι or PKCζ significantly reduces pancreatic cancer cell anchorage-independent growth and cellular invasion [2, 3]. Since PKCι and PKCζ share significant homology (85% homologous in the catalytic domains [15]), one interpretation of this observation is that the atypical PKC isozymes play redundant roles in the transformed growth of pancreatic cancer cells. We therefore evaluated the ability of PKCι to reconstitute the effects of PKCζ knockdown, and PKCζ to reconstitute the effects of PKCι knockdown, on the transformed phenotype of pancreatic cancer cell lines. Panc-1 cells expressing control NT RNAi, PKCι RNAi or PKCζ RNAi were co-transfected with empty (control) vector, or vector expressing PKCι or PKCζ (Figure 1A). As expected, cells expressing PKCι RNAi or PKCζ RNAi formed significantly fewer colonies in soft agar than cells expressing NT RNAi (Figure 1B). Similarly, cells expressing PKCι RNAi or PKCζ RNAi exhibited significantly reduced invasion through matrigel in a transwell invasion assay, compared to Panc-1 cells expressing NT RNAi (Figure 1C). Interestingly, expression of exogenous PKCι, but not PKCζ, was able to significantly reconstitute the effects of PKCι RNAi on pancreatic cancer cell growth in soft agar and invasion (Figure 1B and 1C). Conversely, expression of exogenous PKCζ, but not PKCι, was able to significantly reconstitute the effects of PKCζ RNAi on pancreatic cancer cell growth in soft agar and invasion (Figure 1B and 1C). These data indicate that PKCι and PKCζ play non-redundant roles in the maintenance of the transformed phenotype of pancreatic cancer cells. Consistent with this observation, we have shown that PKCι and PKCζ are preferentially coupled to distinct cancer-promoting signaling pathways in pancreatic cancer cells [2, 3]. Thus we hypothesized that inhibition of expression of both PKCι and PKCζ would reduce the transformed phenotype significantly more than inhibition of either aPKC alone. To test this hypothesis, Panc-1 cells were co-transfected with NT and PKCι RNAi, NT and PKCζ RNAi, or PKCι RNAi and PKCζ RNAi (Figure 1D), and assessed for anchorage independent growth and cellular invasion. As predicted, cells expressing both PKCι and PKCζ RNAi produced significantly fewer colonies in soft agar (Figure 1E) and were significantly less invasive (Figure 1F) than NT, PKCι or PKCζ RNAi cells. Taken together, these data demonstrate that PKCι and PKCζ function in a non-redundant way to promote the transformed phenotype of pancreatic cancer cells.

### Par6 knockdown phenocopies aPKC knockdown in pancreatic cancer cells *in vitro*

**Figure 2:**
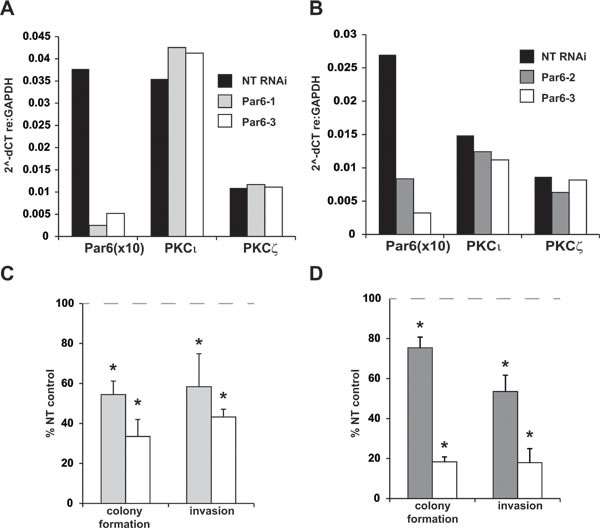
Par6 knockdown phenocopies the effect of aPKC knockdown on pancreatic cancer cell transformed growth and invasion Panc-1 (**A**) and MIA PaCa-2 (**B**) cells stably expressing either NT or Par6-targeted RNAi constructs were evaluated for Par6, PKCι, and PKCζ mRNA expression (PKC expression presented as 2^-dCT, Par6 expression =2^-dCT x 10 for presentation on same scale). **C, D**) The effect of Par6 knockdown on anchorage-independent growth (soft agar colony formation) and cellular invasion was assessed in (**C**) Panc-1 and (**D**) MIA PaCa-2 cells. Bar color indicates Par6-targeting RNAi construct utilized, corresponding to the labels in A, B). Results are expressed as percent NT control±SD n=3, and graph is representative of 2 or more independent experiments. *p < 0.05 *vs* NT.

Par6 is a PB1 domain-containing protein that interacts with aPKCs via PB1 domain-mediated interactions [16]. The Par6-aPKC interaction is necessary for the establishment and maintenance of the cell polarity complex in non-transformed cells [17]. Furthermore, the PB1 domain-mediated interaction of Par6 and PKCι is required for PKCι oncogenic signaling in transformed cells [4, 6, 18]. If the functional effects of PKCι and PKCζ in pancreatic cancer cells require PB1 domain-mediated interactions, then Par6 may also be required for the transformed phenotype of pancreatic cancer cells. To assess the role of Par6 in pancreatic cancer cells, Panc-1 and MIA PaCa-2 cells were stably transfected with control (NT) RNAi or Par6-targeting RNAis (Figure 2A, Panc-1; 2B, MIA PaCa-2). Similar to the effect of knockdown of PKCι or PKCζ, pancreatic cancer cells expressing Par6 RNAi exhibit significantly reduced anchorage independent growth and cellular invasion, compared to cells expressing NT RNAi (Figure 2C, Panc-1; Figure 2D, MIA PaCa-2). These data indicate that oncogenic signaling of PKCι and PKCζ requires PB1 domain-mediated interactions with Par6 and also suggest that a small molecule inhibitor targeting the PB1 domain interactions of aPKCs will significantly reduce pancreatic cancer cell colony formation and invasion.

### ATM inhibits aPKC signaling *in vitro*

**Figure 3:**
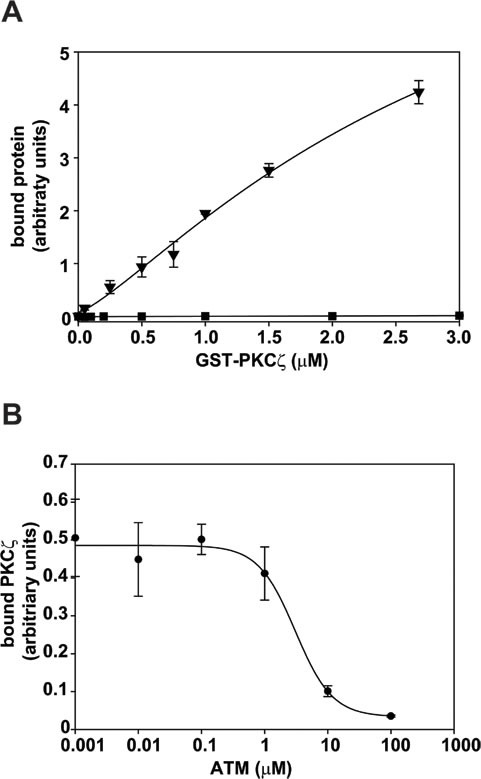
ATM inhibits PB1 domain-mediated interactions (**A**) Binding of GST-PKCζ (1-113, ▼) or GST (■) to Par6. Biotinylated Par6 was bound to streptavidin-coated plates and incubated with increasing concentrations of purified protein. Bound peptide was detected using the MesoScale Discovery detection system. (**B**) ATM inhibits binding of GST-PKCζ to Par6. Biotinylated Par6 bound to streptavidin-coated plates was incubated with 0.4μM of GST-PKCζ in the presence of increasing concentrations of ATM. The average of 3 replicates±SD is plotted and graph is representative of 2 or more independent experiments.

A high throughput screen of FDA-approved drugs identified small molecule inhibitors of PKCι-Par6 PB1-domain interactions [6]. ATM, a gold-containing compound identified with this screen, significantly inhibits PKCι-mediated transformed growth of lung cancer cells, *in vitro* and *in vivo* [4, 9, 19]. Gold-containing compounds are known to be thio-reactive, covalently modifying proteins by formation of thio-gold adducts with reactive cysteine residues [20]. ATM selectively modifies the Cys69 residue within the OPCA motif of the PKCι PB1 domain and disrupts the PKCι-Par6 PB1 domain interaction in a dose-dependent manner [6]. Interestingly, a similar cysteine residue is found in the PB1 domain of PKCζ (Cys68), but not in any other known PB1 domains [15]. The PB1 domain of PKCζ binds Par6 in a specific manner (Figure 3A). As predicted by the presence of the ATM-modifiable cysteine within the PKCζ PB1 domain, ATM also inhibits the binding of the PB1 domain of PKCζ to Par6 in a dose-dependent manner (Figure 3B), with an IC50 value (3.0 μM) similar to the IC50 value calculated for disruption of PKCι-Par6 PB1 domain interaction [6].

**Figure 4:**
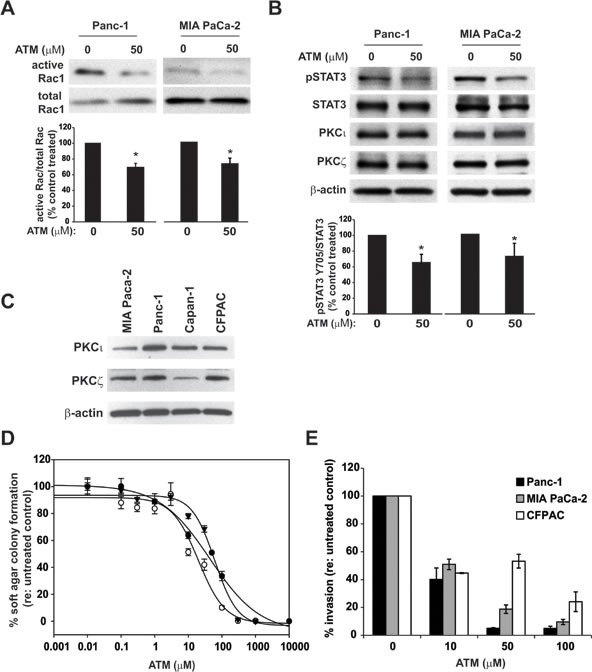
ATM inhibits aPKC downstream signaling and transformed growth in pancreatic cancer cell lines **A**) Panc-1 and MIA PaCa-2 cells were pre-treated with ATM (50 μM) for 48hrs then assayed for Rac1 activity. Immunoblot analysis of Rac1 in precipitates (active Rac1) and total cellular extracts (total Rac1) is shown. The ratio of active Rac1/total Rac1 is plotted relative to control-treated cells (±SD, n=4 independent experiments *p < 0.05). **B**) Panc-1 (left) and MIA PaCa-2 (right) cells were treated with ATM (50 μM) for 48hrs then subject to immunoblot analysis for expression of p-STAT3, total STAT3, PKCι, PKCζ, and β-actin as a loading control. The ratio of active p-STAT3/total STAT3 is plotted relative to control-treated cells (±SD, n=4 independent experiments *p < 0.05). **C**) Immunoblot analysis of PKCι, PKCζ and β-actin expression in MIA PaCa-2, Panc-1, Capan-1 and CFPAC pancreatic cancer cell lines. **D**) Anchorage-independent growth was assessed in Panc-1 (open circles), MIA PaCa-2 (triangle), and Capan-1 cells (filled circle) in the presence of increasing concentrations of ATM (μM). Bar=average of at least 4 replicates±SD. **E**) Cellular invasion was assessed in Panc-1, MIA PaCa-2, and CFPAC cells pre-treated for 48hrs with ATM and in the presence of increasing concentrations of ATM (μM). Bar=average of 3 replicates±SD. Each panel is representative of 2 or more independent experiments.

PKCι, PKCζ and Par6 are required for pancreatic cancer cell transformed growth; thus, we hypothesized that molecular inhibition of aPKC PB1-domain-mediated interactions will inhibit the transformed signaling of both PKCι and PKCζ. Panc-1 and MIA PaCa-2 pancreatic cancer cell lines were treated with ATM for 48hrs and then assayed for Rac1 activity. ATM treatment inhibits Rac1 activity in pancreatic cancer cells (Figure 4A) consistent with inhibition of PKCι signaling [2]. Additionally, ATM inhibits STAT3 activation, as detected by a significant decrease in phosphorylation of Y705 (Figure 4B), consistent with inhibition of PKCζ signaling [3]. These data indicate that ATM inhibits pancreatic cancer oncogenic signaling pathways regulated by both PKCι and PKCζ *in vitro*.

### Pharmacological inhibition of aPKCs inhibits the transformed phenotype of pancreatic cancer cells *in vitro*

**Figure 5:**
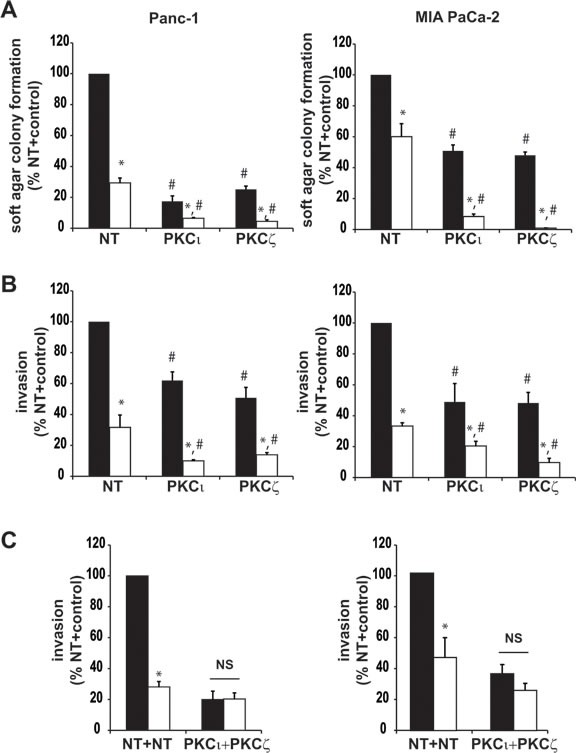
Effect of aPKC knockdown on pancreatic cancer cell sensitivity to ATM inhibition of the transformed phenotype **A**) Anchorage-independent growth was assessed in Panc-1 (left), and MIA PaCa-2 (right), cells stably expressing NT, PKCι, or PKCζ RNAi in the presence of control diluent (filled bars) or 50 μM of ATM (open bars). Bar=average of at least 4 replicates±SD. **B**) Cellular invasion was assessed in Panc-1 (left), and MIA PaCa-2 (right), cells stably expressing NT, PKCι, or PKCζ RNAi in the presence of control diluent (filled bars) or 50 μM of ATM (open bars). Bar=average of 3 replicates±SD. *p < 0.05 versus the same RNAi construct with control treatment, #*p* < 0.05 versus NT RNAi with same treatment. **C**) Cellular invasion was assessed in Panc-1 (left), and MIA PaCa-2 (right), transfected with two doses of NT RNAi (control), or PKCι and PKCζ RNAi, in the presence of control diluent (filled bars) or 50 μM of ATM (open bars). Bar=average of 3 replicates±SD, *p < 0.05 versus the same RNAi construct with control treatment, NS=difference is not statistically significant. Each panel is representative of 2 or more independent experiments.

We have previously shown that knockdown of the aPKCs inhibits the transformed phenotype of pancreatic cancer [2, 3]; therefore we predicted that a pharmacological inhibitor of aPKC signaling would mimic genetic inhibition. PKCι and PKCζ are both expressed in multiple human pancreatic cancer cell lines (Figure 4C), corroborating our published data demonstrating high expression of aPKCs in pancreatic cancer patient tumors [2, 3]. Panc-1, MIA PaCa-2, and Capan-1 pancreatic cancer cell lines were grown in soft agar in the presence of increasing concentrations of ATM (Figure 4D). ATM inhibited anchorage-independent growth of pancreatic cancer cell lines in a dose-dependent manner. Additionally, ATM inhibited pancreatic cancer cell invasion in a dose-dependent manner (Figure 4E). Recently, another thio-gold-containing compound, ANF, has been shown to also inhibit PKCι signaling by a mechanism similar to ATM [7]. As predicted, ANF also inhibited anchorage-independent growth (Figure S1A) and invasion (Figure S1B) of pancreatic cancer cell lines in a dose-dependent manner. These data indicate that clinically relevant therapies targeting aPKCs can inhibit the transformed phenotype of pancreatic cancer cells,

We hypothesized that the effect of ATM on the transformed phenotype of pancreatic cancer cells is due to inhibition of both PKCι and PKCζ. We therefore predicted that pancreatic cancer cells expressing either PKCι or PKCζ RNAi would retain sensitivity to ATM-mediated inhibition of anchorage-independent growth and invasion. As predicted, ATM treatment of pancreatic cancer cells expressing PKCι RNAi further reduced anchorage-independent growth (Figure 5A) and cellular invasion (Figure 5B). Likewise, ATM further reduced anchorage-independent growth (Figure 5A) and invasion of pancreatic cancer cells expressing PKCζ RNAi (Figure 5B). Furthermore, combined knockdown of PKCι and PKCζ by RNAi inhibited invasion to a level similar to the effect of ATM treatment (Figure 5C); these cells were insensitive to additional inhibition by ATM treatment, indicating that PKCι and PKCζ are the primary cellular targets for ATM-mediated inhibition (Figure 5C).

### ATM inhibits pancreatic orthotopic tumor growth, aPKC regulated signaling and metastasis *in vivo*

**Figure 6:**
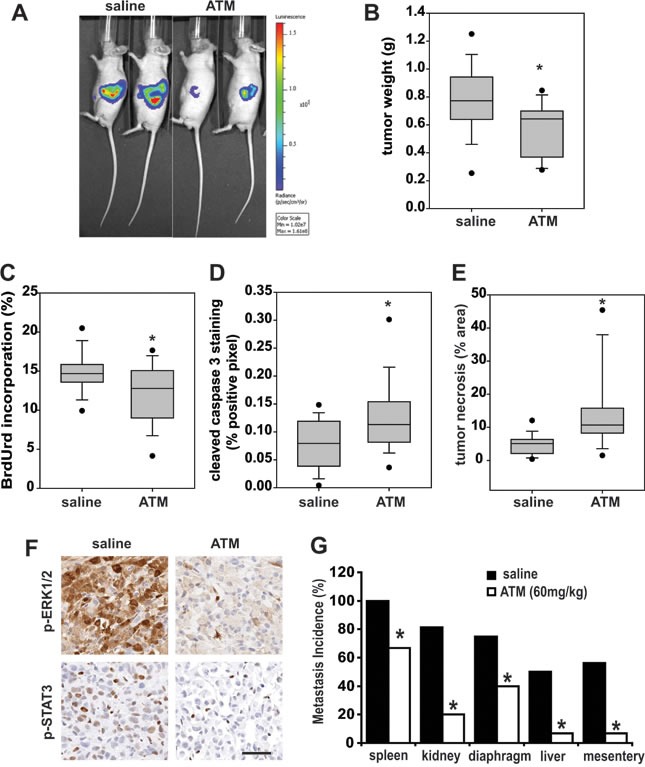
ATM inhibits Panc-1 tumor size and aPKC signaling pathways *in vivo* **A**) Tumor growth was monitored by bioluminescence (total flux, photons per second) detected by IVIS imaging of orthotopic Panc-1 pancreatic tumors in live, anesthetized mice. Representative bioluminescent imaging of mice with orthotopic Panc-1 pancreatic tumors treated daily with either saline or 60mg/kg ATM is shown. Mice were sacrificed after 4 weeks of treatment and tumors were analyzed for the following characteristics: **B**) final tumor weight, **C**) tumor BrdUrd incorporation, **D**) cleaved caspase-3, and **E**) tumor necrosis as described in Materials and Methods. **F**) Mice carrying orthotopic Panc-1 tumors were treated with daily injections of saline or 60mg/kg ATM for 7 days and harvested 24 hours after the final injection. Tumors were assessed for p-ERK1/2 (top panels), and p-STAT3 (bottom panels) by IHC staining. Bar=100 μm. **G**) Mice treated for 4 weeks with ATM or saline were evaluated for metastases to distal organs as described in Materials and Methods. The percentage of mice in each treatment group with confirmed metastasis to each distal organ is plotted, n = 16 per group, Difference in metastasis to each organ was analyzed by Fisher Exact test and *p < 0.05 was considered significant.

We next investigated the effect of ATM treatment on pancreatic tumor growth using a previously described Panc-1 orthotopic tumor model [2]. Panc-1 cells expressing the firefly luciferase gene (pSIN-Fluc) were injected into the pancreas of nude mice and tumor growth was monitored by bioluminescence (Figure 6A). Mice were treated with either 60mg/kg ATM or saline by daily IP injections for 5 weeks. Tumor formation was observed in all mice; however, mice treated with ATM had significantly lower final tumor weight compared to those treated with saline (Figure 6A and 6B). Tumor cell proliferation was also significantly reduced in tumors from ATM-treated mice (Figure 6C). Additionally, tumors from ATM-treated mice had a significant increase in apoptosis (Figure 6D), and tumor necrosis (Figure 6E). These data indicate that the reduced tumor size in ATM-treated mice is due to a combination of decreased tumor cell proliferation and increased cell death. p-ERK1/2 and p-STAT3 levels were reduced in tumors from ATM-treated mice (Figure 6F), consistent with the effects of inhibition of PKCι [2], and PKCζ [3], respectively, demonstrating target engagement *in vivo*. Furthermore, significantly fewer metastases to distal organs were detected in mice treated with ATM compared to saline-treated mice (Figure 6G). These data demonstrate that pharmacological inhibition of aPKCs is a promising therapeutic approach for the treatment of pancreatic cancer.

## DISCUSSION

We have previously shown that both PKCι and PKCζ play promotive roles in the transformed growth of pancreatic cancer cells *in vitro* and *in vivo* [2, 3]. In the present study, we first demonstrate that PKCι and PKCζ play non-redundant roles in pancreatic cancer, and that knockdown of both PKCι and PKCζ results in significant, additive inhibition of the transformed phenotype compared to inhibition of either aPKC alone. These data extend our previous observations that PKCι and PKCζ are preferentially coupled to distinct pro-cancer signaling pathways in pancreatic cancer [2, 3]. PKCι promotes the transformed phenotype of pancreatic cancer, at least in part, via activation of Rac1-MEK/ERK signaling [2], whereas a major mechanism by which PKCζ regulates pancreatic cancer cell transformed growth is via promotion of STAT3 activation [3]. Therefore, an inhibitor targeting both aPKCs would simultaneously inhibit two critical oncogenic signaling pathways in pancreatic cancer.

In addition to pancreatic cancer, PKCι has been shown to play an oncogenic/cancer-promoting role in lung, ovarian, and colon cancers (reviewed in Murray et al. [21]). The oncogenic role of PKCι has been particularly well-described in lung cancer, where PKCι is required for oncogenic K-ras-induced tumor initiation and progression in a mouse model of lung cancer, as well as for maintenance of the transformed phenotype of human lung cancer cells [5, 18, 22]. Further investigation of the mechanism of PKCι oncogenic signaling in lung cancer revealed that PKCι mediates its effects by forming an oncogenic signaling complex through interactions involving its PB1-domain [4, 9, 19].

A high throughput screen of a library of FDA-approved drugs for compounds that disrupted the PB1 domain-mediated interaction between PKCι and Par6 identified several gold-containing compounds, used clinically for the treatment of rheumatoid arthritis [6]. One of the gold-containing drugs identified as a PKCι PB1 domain interaction disruptor, ATM, inhibits lung cancer cell transformed growth *in vitro* and *in vivo* [6, 19]. FDA-approved gold-containing small molecules have been used clinically for over 30 years for the treatment of rheumatoid arthritis [23]. While the mechanism of action of gold-containing drugs in the treatment of rheumatoid arthritis has never been fully elucidated, the inhibition of mitochondrial thioredoxin reductase has been suggested as a mediator of therapeutic response [20]. Thioredoxin reductase is considered an important target for immunosuppression in the treatment of rheumatoid arthritis, since thioredoxin reductase is highly expressed in the synovial cells of rheumatoid arthritis patients [24]. Other hypothesized mechanisms of action of gold-containing compound-induced immunosuppression are inhibition of IL-6/JAK/STAT3 signaling and inhibition of NF-κB [25-27]. Recent evaluation of gold-containing rheumatoid arthritis drugs for anti-cancer properties has revealed both apoptotic and cytotoxic effects in various cancer cell types, including breast, ovarian and leukemia [28-31]. These studies investigated multiple potential targets for the anti-cancer effects of these drugs, including inhibition of thioredoxin reductase, STAT3 signaling, proteasome-associated deubiquitinases and telomerase [28-31]. While the mechanism of action of gold-containing drugs in most cancer types remains poorly understood, mechanistic studies in lung cancer cells demonstrate that ATM-mediated inhibition of transformed growth requires the gold-modifiable Cys69 residue in the PKCι PB1 domain, demonstrating the necessity for PKCι inhibition for the anti-tumor effects of ATM in lung cancer cells [4, 6, 19]. Furthermore, sensitivity to ATM-mediated growth inhibition in lung cancer cells correlates directly with expression of PKCι and Par6 but not to other proposed ATM targets such as thioredoxin reductases [19].

**Figure 7:**
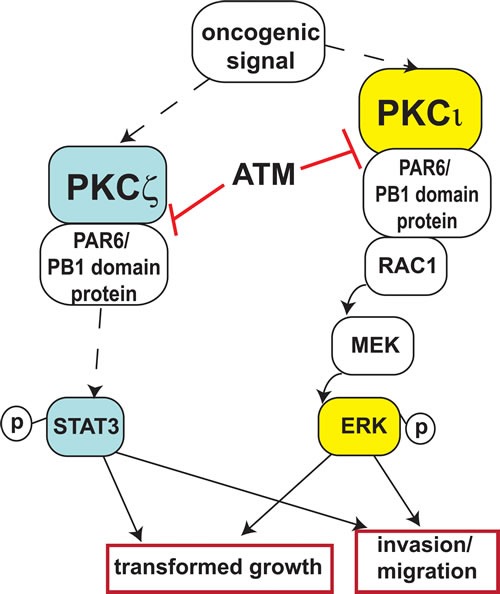
Summary of ATM-mediated inhibition of aPKC signaling

Similar to PKCι, the PB1 domain of PKCζ contains a cysteine residue at position 68; thus we hypothesized that ATM would disrupt PKCζ PB1 domain-mediated interactions in a similar manner to its effect on PKCι-Par6 interactions [4, 16]. Indeed, *in vitro* analysis demonstrated that ATM disrupts the interaction of the PKCζ PB1 domain and Par6 with an IC50 value similar to that characterized for a PKCι-Par6 interaction [4, 6]. We next evaluated the therapeutic potential of pharmacological inhibition of both PKCι and PKCζ in pancreatic cancer cells. ATM inhibited both PKCι- and PKCζ-regulated signaling pathways in pancreatic cancer cells *in vitro* and *in vivo*. Likewise, ATM inhibited the transformed growth of pancreatic cancer cells *in vitro* and *in vivo*, similar to the effect of genetic inhibition of PKCι and PKCζ. These data demonstrate that ATM is able to simultaneously target both PKCι and PKCζ, thereby inhibiting two critical pathways in pancreatic cancer (Figure 7).

In addition to ATM, ANF, a gold-containing drug used to treat rheumatoid arthritis, has been evaluated for anti-cancer activity. ANF was shown to inhibit PKCι signaling in ovarian cancer cells, and inhibit PKCι-mediated transformed growth, similar to the effect of ATM in lung cancer cells [4, 7]. Previously published structure-activity studies of gold (I) complexes demonstrated that derivatives containing a phosphine group (ANF but not ATM) have increased cytotoxic effects against cancer cells [32], and that *in vitro* cytotoxicity and *in vivo* antitumor effects are related to the increased lipophilicity provided by the phosphine group [33]. Likewise, we observed that ANF inhibited anchorage-independent growth and invasion in pancreatic cancer cells with a greater potency than ATM (Figure 4 and Figure S1). Taken together, we show that gold-containing compounds inhibit the transformed growth of pancreatic cancer cell lines by inhibiting PKCι- and PKCζ-regulated signaling pathways. These data further support the therapeutic potential of targeting the aPKCs in pancreatic cancer.

Unlike several other cancers, the lethality of pancreatic cancer has gradually increased over the past decade [1] and thus the need for more successful treatments has grown more critical. Our previous work identified the aPKCs, PKCι and PKCζ, as potential therapeutic targets for the treatment of pancreatic cancer. ATM and ANF have been shown to specifically inhibit PKCι signaling and tumor growth in lung [6, 19] and ovarian cancer models [7]. Recently, ATM was evaluated in a phase I clinical trial of patients with advanced non-small cell lung cancer, ovarian, and pancreatic cancers [34]. This study identified a maximum tolerated dose (MTD) for ATM corresponding to serum gold levels above the level necessary to successfully inhibit aPKC signaling and reduce tumor growth in preclinical models, indicating the clinical relevance of ATM and gold-containing compounds for the treatment of cancers in which aPKC signaling promotes the cancer phenotype [19, 34]. In the present study, we provide pre-clinical evidence that ATM disrupts aPKC signaling and inhibits the transformed phenotype in pancreatic cancer, and demonstrate that aPKCs are the major target of ATM-mediated inhibition of the transformed phenotype. Therefore aPKC inhibitors may be a promising new treatment option for pancreatic cancer patients. Future clinical studies are necessary to determine the efficacy of ATM and ANF for the treatment of pancreatic cancer.

## MATERIALS AND METHODS

### Reagents and cell culture

Human pancreatic cancer cell lines were purchased from American Type Culture Collection and all experiments were performed with cells passaged less than 6 months. Human pancreatic cancer cell lines were maintained in a 5% CO_2_ humidified tissue culture incubator in DMEM (Panc-1, MIA PaCa-2, and CFPAC) or IMDM (Capan-1) with 10% (fetal bovine serum) FBS as recommended by American Type Culture Collection. Antibodies were obtained from the following sources: PKCζ, β-actin, phospho-STAT3 (Y705), STAT3, phospho-ERK1/2, ERK1/2 and cleaved caspase-3 (all from Cell Signaling Technologies), PKCι (BD Transduction Laboratories), and 5-bromo-2′-deoxyuridine (BrdUrd) (DakoCytomation). GST-PAK1-p21 binding domain (PBD) beads were generated as previously described [8]. Briefly, BL21 bacteria harboring the fusion protein GST-human PAK1 PBD in the pGEX2TK vector (received from Howard Crawford) were induced with IPTG then lysed, purified, and mixed with Glutathione Sepharose 4B beads. ATM was purchased from Akon Pharmaceuticals and Sigma, ANF was purchased from Santa Cruz Biotechnology, Inc.

### RNA isolation, quantitative real-time PCR (qPCR) and analysis

Total RNA was isolated using RNAqueous Isolation Kit (Ambion) according to the manufacturer's protocols. TaqMan® Gene Expression Assay primer and probe sets (Applied Biosystems) were used for real-time, quantitative PCR (qPCR) analysis of GAPDH (Hs99999905_m1), PKCζ (Hs00177051_m1), Par6 (Hs00180947_m1) and 18S (Hs99999901_s1). Forward and reverse primer and probe sequences for PKCι were previously described [2]. qPCR analysis was carried out using 10 ng of cDNA or 2 ng cDNA (18S) on an Applied Biosystems 7900 thermal cycler. Data was evaluated using the SDS 2.3 software package. Gene expression was normalized to GAPDH. All data is expressed as 2^−(*CT*(target)−*CT*(endogenous reference))^.

### Knockdown and re-expression of human PKCι and PKCζ genes

Lentiviral vectors carrying short hairpin RNA interference (RNAi) constructs targeting PKCι, PKCζ, or Par6 were generated and used to obtain stable transfectants as described previously [3, 9]. PKCι RNAi construct - GCCTGGATACAATTAACCATT; PKCζ RNAi construct - GTTGTTCCTGGTCATTGAGTA; Par6 RNAi constructs – TCAGTCATAGACGTGGACCTA (Par6-1), TGGACGTGCTACTTGGCTATA (Par6-2) and GCTGAGCCTGATAGTGACGAT (Par6-3). The cDNA for human PRKCZ (NM_002744.4) was cloned into the pBABE retroviral expression vector. Cells were stably transfected with pBabe, pBabe-PKCι [10] or pBabe-PKCζ. Cell populations carrying the viral constructs were selected and maintained by inclusion of puromycin in the culture media.

### Anchorage-independent growth assays

Pancreatic cancer cells (7×10^3^) were plated in soft agar and assessed for anchorage-independent growth as described previously [2, 3].

### Cellular invasion assay

Cellular invasion was assayed using Matrigel-coated invasion chambers (BD Biosciences) according to the manufacturer's protocol. Briefly, 5×10^4^ human pancreatic cancer cells were plated in serum-free media in the top chamber, and media containing 2.5% FBS was used as the chemo-attractant in the bottom chamber. Cells were allowed to invade for 18hrs at 37°C and then cells were fixed, stained and quantitated as previously described [11].

### PKCζ and Par6 binding assay

Streptavidin-coated 96-well singleplex plates (MesoScale Discovery, Rockville, MD) were blocked with 5% non-fat dry milk for 1hr at room temperature. Wells were washed twice with PBS/Tween. After washing, recombinant biotinylated human Par6 protein was bound by incubation at room temperature for 1 hr. GST-tagged PKCζ N-terminal fragment (amino acids 1-113) or GST control were purified via B-PER GST Protein Purification Kit (Pierce, Rockford, IL) and labeled with Sulfo-Tag NHS-Ester for 2 hrs. After removing the free Sulfo-Tag NHS-Ester reagents, increasing concentrations of purified protein was added to Par6 coated plates. After overnight incubation at 4°C, the wells were washed once with PBS/Tween and 150μl of 1x Reading Buffer was added. Binding to Par6 was determined by detection of electrochemiluminescent signal emitted from the Sulfo-Tag (620 nm), using a SECTOR Imager 2400 (Meso Scale Discovery). To determine the effect of ATM on PKCζ binding to Par6, 0.4μM of GST-tagged PKCζ (1-113) was added to each well along with ATM to achieve the desired final concentration as indicated in the figure, and binding detected as described.

### Rac1 activity assay

Pancreatic cancer cells treated with saline or ATM were harvested and assayed for the presence of active, GTP-bound Rac1. Rac1-GTP was precipitated from cell extracts with PAK-1 PBD agarose beads as described previously [10].

### Orthotopic tumor model

Panc-1 human pancreatic cancer cells (1 × 10^6^) carrying the pSIN-Fluc luciferase expression vector were mixed with growth factor-reduced Matrigel (Becton Dickinson) and injected into the proximal pancreas (n=15 mice/group respectively) of 4-6 week old athymic nude mice. All surgeries were performed under isoflurane anesthesia, and mice were administered buprenorphine as an analgesic immediately before and ~18 hours after the surgery to minimize animal discomfort. Tumor-bearing mice were monitored daily for signs of distress and twice weekly for weight loss. Mice received IP injections of saline or 60mg/kg ATM 5 days/week, beginning on day 8 and continuing until one day prior to sacrifice. For weekly imaging, mice were injected intraperitoneally (IP) with 150 mg/kg body weight D-Luciferin solution (Xenogen), anesthetized with isoflurane and imaged using a bioluminescence imaging system (IVIS Imaging Spectrum System). Bioluminescence was calculated using IVIS Imaging Spectrum software. Week 1 bioluminescence was used to distribute mice evenly between saline and ATM treatment groups. One hour prior to sacrifice, mice were injected IP with 100μg/g BrdUrd. All of the animal experiments were approved by the Mayo Clinic Institutional Animal Care and Use Committee.

### Orthotopic tumor analysis

Formalin-fixed pancreatic tumors were analyzed by immunohistochemical (IHC) detection of BrdUrd incorporation, as described previously [12-14]. Orthotopic pancreatic tumors were evaluated for apoptosis by IHC detection of cleaved caspase-3 as previously described [14]. Tumor necrosis was identified in H&E stained tissue as described previously [3]. Tumors were fixed by perfusion with 10% paraformaldehyde at the time of harvest for detection of expression of p-ERK1/2 and p-STAT3 by IHC analysis. p-ERK1/2 and p-STAT3 staining was visualized using the Envision Plus Anti-Rabbit Labeled Polymer-HRP (Dako). Images were captured and analyzed using Aperio Spectrum software. Staining with only the secondary antibody served as a negative control. Tumor metastases were identified by detection of bioluminescence by ex vivo IVIS imaging of isolated organs and confirmed by examination of H&E stained tissue sections of the distal organs.

### Statistical analysis

Two-way ANOVA, Student *t*-test or Fisher Exact (tumor metastasis) were used to evaluate the statistical significance of the results. *p* < 0.05 was considered statistically significant.

## SUPPLEMENTARY MATERIAL FIGURE



## Abbreviations

ANF: Auranofin
ATM: Aurothiomalate
BrdUrd: 5-bromo-2′-deoxyuridine
H&E: hematoxylin and eosin
IHC: immunohistochemical analysis
PKC: protein kinase C
qPCR: quantitative real time polymerase chain reaction
RNAi: RNA interference
STAT3: Signal transducer and activator of transcription 3
