# Supplementary material for: A small molecule inhibitor of atypical protein kinase C signaling inhibits pancreatic cancer cell transformed growth and invasion
Source: Oncotarget. 2015 Apr 14;6(17):15297–310. doi: 10.18632/oncotarget.3812 (PMC4558152; doi:10.18632/oncotarget.3812)
Supplement: Supplementary file 1 [file oncotarget-06-15297-s001.pdf]

A small molecule inhibitor of atypical protein kinase C signaling inhibits pancreatic cancer cell transformed growth and invasion

Supplementary Material

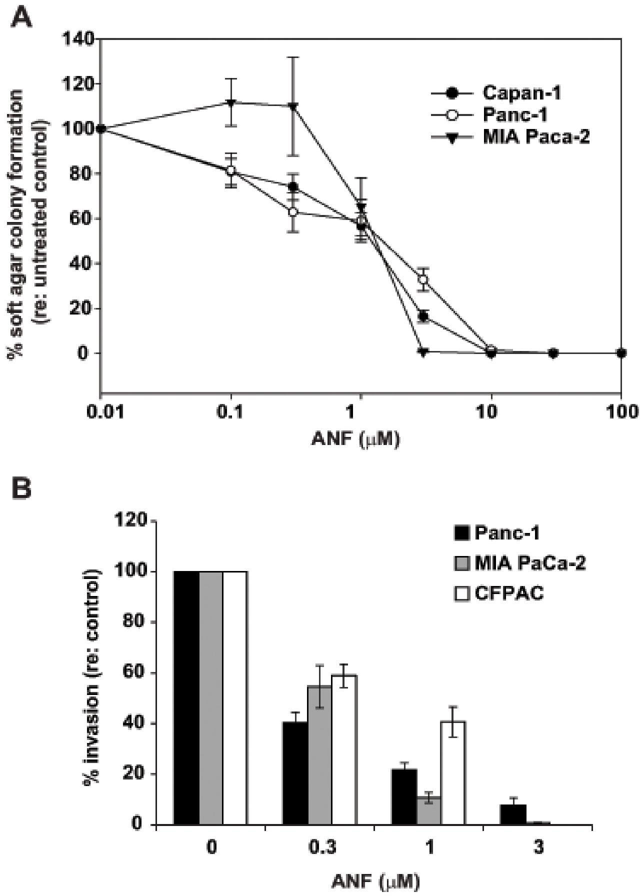

Supplemental Figure 1: ANF inhibits the transformed phenotype of pancreatic cancer cells *in vitro*. A) Anchorage-independent growth was assessed in Panc-1, MIA PaCa-2, and Capan-1 cells in the presence of increasing concentrations of ANF (μM). Bars=average of at least 4 replicates±SD. B) Cellular invasion was assessed in Panc-1, MIA PaCa-2, and CFPAC cells pre-treated for 48hrs with increasing concentrations of ANF (μM), and plotted relative to a DMSO diluent control. Bar=average of 3 replicates±SD. Each panel is representative of 2 or more independent experiments. *=p<0.05
